# PW03-002 – Calculating Gaslini diagnostic score in PFAPA

**DOI:** 10.1186/1546-0096-11-S1-A228

**Published:** 2013-11-08

**Authors:** I Bosio, A Meini, P Cancarini, G Savoldi, M Berlucchi, M Cattalini

**Affiliations:** 1 Pediatric Clinic, University of Brescia, Italy; 2 Laboratorio di Genetica Pediatrica, Anatomia Patologica, Spedali Civili Brescia, Italy; 3 Pediatric Otorynolaryngology, Spedali Civili di Brescia, Brescia, Italy

## Introduction

PFAPA syndrome is the most common cause of periodic fevers in children. In the clinical setting of children with periodic fever, there are a minority of patients in which to differentiate between PFAPA and monogenic periodic fevers is not immediate. The Gaslini score would provide a useful tool in this setting, calculating the probability to carry a mutation for monogenic periodic fevers

## Objectives

we applied the Gaslini score in a cohort of patients with PFAPA syndrome to identify patients with a high Gaslini score. Once stratified our cohort for the Gaslini score, we compared the two populations to study which were the clinical characteristics determining the high risk score.

## Methods

a total of 268 PFAPA patients were recruited and followed in a single referral centre. For the diagnosis Thomas criteria were applied, with the exception of the age at onset criteria. The Gaslini diagnostic score was calculated in each patient.

## Results

Out of the 268 patients considered, 47 had a high Gaslini diagnostic score (>1.32). 30% of these patients were screened for mutations in the MVK, TNFRSF1A, and MEFV genes, but they were all negative. Since the clinical picture of all the remaining high risk patients was very coherent with the PFAPA diagnosis we did not performed any further genetic test. We also compared the two groups: age at the onset, family history, mean duration of the episodes, free interval, clinical features and mean age at resolutions were the variables considered. As expected patients with a high Gaslini diagnostic score had earlier age at onset (*p* = 0.002) and more frequently positive family history (χ^2^ = 0.033). Similarly, the prevalence of abdominal pain and diarrhea was more consistent in patients with high Gaslini score (χ^2^= 0.001) while -unexpectedly- aphthous stomatitis was similar. Cervical adenitis and prodroms were also more frequent in the high score population (χ^2^ = 0.046 and χ^2^= 0.022, respectively).

## Conclusion

The Gaslini score represents a useful tool to identify patients with a high risk of carrying mutations for the monogenic periodic fevers. Even though our results were quite expected, since the differences between the two sub-populations were in the very same parameters considered in the Gaslini score, our study gives a clue on the fact that the score can fail, predicting a higher risk, in young PFAPA patients or in those with positive family history or clinical relevance of abdominal pain. Indeed this is a quite common scenario. Studying our cohort of patients we identified a subgroup of PFAPA children with high Gaslini score, young age at onset, clinical relevance of abdominal pain, cervical adenitis and prodromic symptoms before the fever onset. Whether this is due to a slightly different manifestation of PFAPA -based on the age of onset- or to the presence of two distinct clinical entities within “PFAPA” need to be addressed in future studies.

## Disclosure of interest

None declared

